# Prognostic Value of Reverse Dipper Blood Pressure Pattern in Chronic Kidney Disease Patients not Undergoing Dialysis: Prospective Cohort Study

**DOI:** 10.1038/srep34932

**Published:** 2016-10-07

**Authors:** Cheng Wang, Zengchun Ye, Yan Li, Jun Zhang, Qunzi Zhang, Xinxin Ma, Hui Peng, Tanqi Lou

**Affiliations:** 1 Division of Nephrology, Department of medicine, the Third Affiliated Hospital of Sun Yat-Sen University, Guangzhou, Guangdong 510630, China; 2 Department of Pathology, the First Affiliated Hospital of Sun Yat-Sen University, Guangzhou, Guangdong 510080, China

## Abstract

The “reverse dipping” blood pressure (BP) pattern has been studied among the general population and in individuals suffering from hypertension. However, the prognosis of this pattern in chronic kidney disease (CKD) patients is not known. We monitored BP throughout the day and followed health outcomes in 588 CKD patients admitted to our hospital. Time to all-cause mortality, cardiovascular mortality, renal events and cardiovascular events was recorded. Multivariate-adjusted Cox regression analyses were carried out to detect the prognostic value of a reverse dipping BP pattern. Prevalence of a “dipper”, “non-dipper” and “reverse dippers” was 34.69%, 43.54% and 18.03%, respectively. Patients with a reverse dipping pattern had a higher prevalence of total mortality, cardiovascular mortality, renal events and cardiovascular events than patients with a dipping pattern (*P* < 0.025). Multivariate-adjusted Cox regression analyses showed that reverse dippers (*versus* dippers) were associated with a higher risk of total mortality (hazard ratio [HR], 5.08; 95% confidence interval [CI], 1.79~14.47), cardiovascular mortality (4.17; 1.25~13.88), renal events (3.00; 1.59~5.65) and cardiovascular events (4.12; 1.78~9.51) even after adjustment by 24-h systolic BP. These data suggest that a reverse dipping BP pattern, independent of 24-h levels of systolic BP, has prognostic value in CKD patients not undergoing dialysis.

Chronic kidney disease (CKD) is a public-health issue in China. Prevalence of CKD in China is 10.8%, so the number of patients with CKD in China has been estimated to be ≈119.5 million according to one survey^1^. Hypertension has been reported to be the leading risk factor for death in patients with CKD, and contributes to 45% of deaths in males, and 46% in females in CKD patients^1^,^2^. Prevalence of hypertension in Chinese patients with CKD not undergoing non-dialysis was reported to be 67.3%^3^. Sustained high blood pressure (BP) is an important risk factor that causes deterioration of renal function, and is associated with a high prevalence of cardiovascular events and mortality in CKD patients^4^. Anti-hypertension measures can slow the progression of CKD and cardiovascular disease (CVD) in CKD patients^5^.

Ambulatory blood pressure monitoring (ABPM) is a better predictor of target-organ damage and cardiovascular events than clinical monitoring of BP^6^,^7^,^8^,^9^. ABPM can provide detailed information on BP over a 24-h period. According to the ratio of systolic blood pressure (SBP) at night and day based on ABPM data, patients can be classified as “extreme dippers”, “non-dippers”, “reverse dippers” and “dippers”^10^,^11^,^12^. One study demonstrated that 80% of participants with hypertensive kidney disease had a non-dipping (41%) or reverse dipping (39%) BP profile, and that non-dippers and reverse dippers had more severe damage to target organs (proteinuria and left-ventricular hypertrophy) compared with patients with a normal BP pattern^13^.

However, data on the prognostic role of a reverse dipping BP pattern on all-cause and cardiac mortality in CKD patients not undergoing dialysis are scarce. One study from Italy suggested that CKD patients not undergoing dialysis with a reverse dipping BP pattern carried a greater risk of renal disease and CVD^7^. In our previous observational study, we reported (for the first time) that 21.9% of CKD patients were reverse dippers, 42% of patients were non-dippers and 36.1% were dippers. Reverse dippers had the worst renal function and most severe cardiovascular damage among these patients^14^. Studies on the effect of a reverse dipping BP pattern on the prognosis of Chinese CKD patients not undergoing dialysis are lacking. In this prospective cohort study, 588 Chinese CKD patients not undergoing dialysis were enrolled and followed prospectively. Long-term effects of a reverse dipping BP pattern on the prognosis of these patients were assessed.

## Results

### Baseline Characteristics of the Study Population

**Figure 1:**
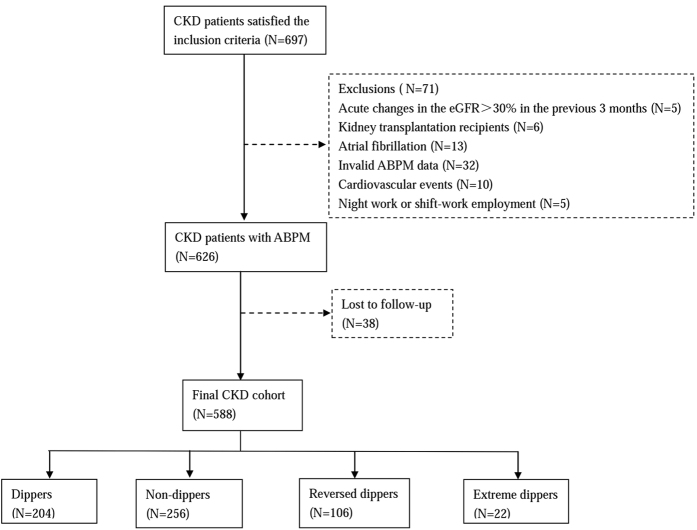
Patient selection and assignment to different blood pressure patterns.

Of the 588 CKD patients enrolled: 397 patients had chronic glomerulonephritis; 63 cases had diabetic nephropathy; 32 subjects had hypertensive nephropathy; 28 individuals had lupus nephritis; and 68 patients had other causes of renal disease (Fig. 1).

Compared with dippers, reverse dippers were older and had a: higher prevalence of diabetes mellitus (DM); higher levels of blood urea nitrogen (BUN) and creatinine; lower levels of hemoglobin and estimated glomerular filtration rate (eGFR); higher levels of proteinuria; higher 24-h SBP, 24-h diastolic blood pressure (DBP), daytime SBP, nighttime SBP and nighttime DBP (*P* < 0.017). Compared with dippers, non-dippers had: higher levels of BUN, intact parathyroid hormone (iPTH) and homocysteine; lower levels of hemoglobin; higher levels of proteinuria; higher 24-h SBP, nighttime SBP and nighttime DBP (*P* < 0.017). Extreme dippers had lower nighttime SBP and nighttime DBP compared with dippers (*P* < 0.017) (Table 1).

### Incidence of events

With regard to total mortality, median follow-up was 35 (interquartile range (IQR): 24–49) months, and total follow-up amounted to 1762 patient-years. During follow-up, 44 patients died (event incidence: 24.98 per 1000 patient-years). Causes of death were: fatal heart failure (22 patients); acute myocardial infarction (10); stroke (7); malignant tumor (4); gastrointestinal bleeding (1). Hence, 39 patients among this cohort died of cardiovascular events (event incidence: 22.00 per 1000 patient-years).

With regard to renal events, median follow-up was 31 (IQR: 19–45) months, and total follow-up time amounted to 1594 patient-years. During follow-up, 140 renal events were recorded in this cohort (event incidence: 87.83 per 1000 patient-years).

With regard to cardiovascular events, median follow-up was 34 (IQR: 24–49) months, and total follow-up time amounted to 1717 patient-years. During follow-up, 74 cardiovascular events were recorded (event incidence: 43.10 per 1000 patient-years): 39 fatal events (as described above) and 35 non-fatal events (27 heart failures, 7 acute myocardial infarctions, and 1 acute arterial occlusion of lower extremities).

### Risks associated with reverse dippers

Crude and standardized prevalence of total mortality, cardiovascular mortality, renal events and cardiovascular events was higher in reverse dippers than in dippers (*P* < 0.025) (Table 2).

**Figure 2:**
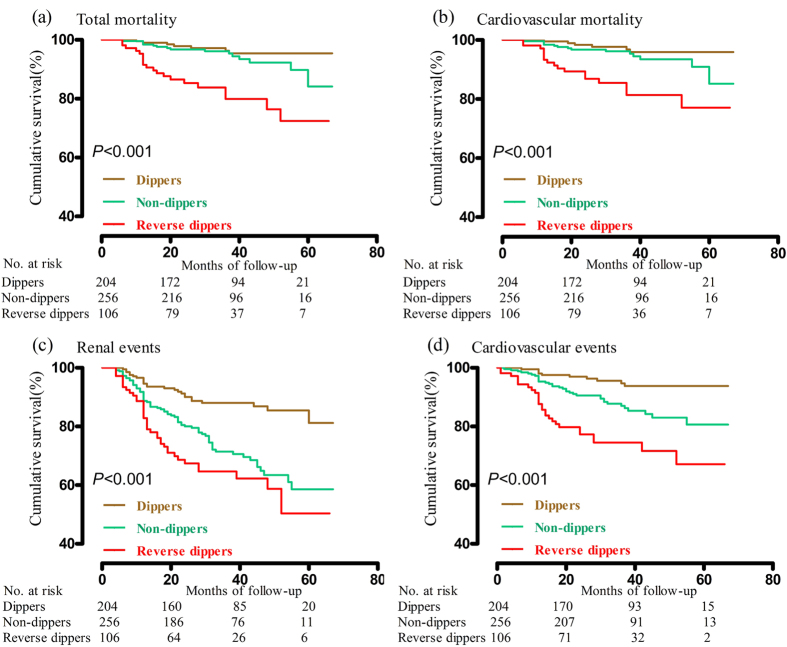
Cumulative survival for total mortality (**a**), cardiovascular mortality (**b**), renal events (**c**) and cardiovascular events (**d**) in patients by blood pressure pattern. *P* values are for the difference among the three groups by log-rank test.

With respect to total mortality, cardiovascular mortality, renal events and cardiovascular events, there was a significant difference among the three survival curves (*P* < 0.001). For all endpoints, survival of reverse dippers was lower than that of dippers (*P* < 0.025) (Fig. 2).

A competing risks model was carried out to identify the hazard ratios. In partly adjusted models, having a reverse dipping BP pattern (*versus* having a dipping BP pattern) was associated with a higher risk of total mortality (hazard ratio [HR], 6.36; 95% confidence interval [CI], 2.30~17.62), cardiovascular mortality (5.39; 1.67~17.42), renal events (3.34; 1.81~6.14) and cardiovascular events (4.75; 2.05~11.00); Also, having a non-dipping pattern (*versus* having a dipping BP pattern) was associated with a higher risk of renal events (1.92; 1.09~3.37). When these models were also adjusted for 24-h BP, having a reverse dipping BP pattern (*versus* having a dipping BP pattern) continued to be associated with a higher risk of total mortality (5.08; 1.79~14.47), cardiovascular mortality (4.17; 1.25~13.88), renal events (3.00; 1.59~5.65) and cardiovascular events (4.12; 1.78~9.51); while non-dippers was no longer associated with prognosis (Table 3).

## Discussion

In this prospective cohort study, 588 CKD patients not undergoing dialysis were enrolled and followed prospectively for a mean duration of 35 months. Patients with a reverse dipping BP pattern showed a worse prognosis compared with patients with a dipping BP pattern. A reverse dipping BP pattern was associated with an increased risk of total mortality, cardiovascular mortality, renal events and all cardiovascular events even when adjusted by 24-h SBP, but the significant association between a non-dipping BP pattern and prognosis disappeared after adjustment by 24-h SBP. Taken together, these results suggest that a reverse dipping BP pattern, rather than a non-dipping BP pattern, independent of 24-h SBP, is a risk factor for the prognosis in Chinese CKD patients not undergoing dialysis. Hence, special attention should be paid to a reverse dipping BP pattern in CKD patients.

Studies have shown that a non-dipping BP pattern is correlated with an increase in target-organ damage and cardiovascular events in hypertensive and normotensive subjects, and this BP pattern has been considered to be an additional risk factor for CVDs^15^,^16^,^17^. Compared with a non-dipping BP pattern, a reverse dipping BP pattern does not involve a fall in BP at night^12^. In a study looking at the role of a reverse dipping BP pattern in non-CKD elderly patients with sustained hypertension, reverse dippers had approximately twice the risk for stroke compared with dippers or non-dippers^18^. A study by Ohkubo T. and colleagues suggested that a decrease in nocturnal BP fall was significantly associated with an increase in the risk of cardiovascular mortality in hypertensive patients^19^. They also found that patients with a normal BP range and diminished nocturnal reductions in BP had an increased risk of cardiovascular mortality^19^. However, few reports on a reverse dipping BP pattern in CKD patients have been carried out. Minutolo *et al*. showed that CKD patients who were non-dippers and those who were reverse dippers had an increased risk of renal-related death and cardiovascular events^7^. Data on the role of a reverse dipping BP pattern in Chinese CKD patients are lacking. We were the first to report that the prevalence of a reverse dipping BP pattern is associated with target-organ damage in Chinese CKD patients, and suggested that reverse dippers have a worse prognosis. We found a reverse dipping BP pattern to be associated with an increased risk of total mortality, cardiovascular mortality, renal events and all cardiovascular events even when adjusted by 24-h SBP. Also, the association between a non-dipping BP pattern and increased risk of total mortality, cardiovascular mortality, renal events and all cardiovascular events was lost after adjustment by 24-h SBP. Hence, we should pay more attention to a reverse dipping BP pattern, rather than a non-dipping pattern, in Chinese CKD patients.

Patients with a reverse dipping BP pattern had nocturnal increases in BP, whereas individuals with a dipping pattern had a nocturnal fall in BP of 10–20%. Hence, the reverse dipping pattern is opposite to the physiologic rhythm of BP. Nocturnal BP represents the minimal BP needed for adequate perfusion of organs in healthy subjects^20^. Maintenance of a high BP at night overloads the cardiovascular system, with a consequent negative impact on the heart and vascular structures. It was not surprising to find that reverse-dipper CKD patients had a worse prognosis. Therefore, lowering nocturnal BP and normalizing the BP pattern might help to reduce the risks of CVD and renal disease for such CKD patients. Antihypertensive chronotherapy could be used to lower nocturnal BP and normalize the BP pattern^21^. Previously, we showed the advantages of bedtime scheduling of valsartan (80–320 mg, once daily for 1 year) in 60 patients with CKD^22^. Further prospective randomized clinical trials are needed to ascertain if antihypertensive chronotherapy has a beneficial effect on CKD patients.

The present study had six main limitations. First, all patients were from a single center. Second, all CKD patients underwent only one ABPM, so we could not rule out changes in subsequent ABPM. Third, patients who are willing to finish their first follow-up were enrolled, whereas patients who refused to complete follow-up were excluded, which led to a bias. Fourth, all patients did not accept standard therapy, so we could rule out the effect of drugs. Fifth, the clinical and significant differences in baseline characteristics between groups, though adjusted by the most common risk factors, might have affected our results. Finally, the etiology of our CKD patients varied. The underlying etiology (e.g., DM) may have affected the BP pattern, which may have led to different results in different CKD patients.

In conclusion, we have provided the first evidence that a reverse dipping BP pattern, independent of 24-h SBP, has prognostic value in Chinese CKD patients not undergoing dialysis. Further, prospective randomized clinical trials are needed to clarify if correction of BP patterns by administration of antihypertensive drugs at night improves the prognosis and attenuates progression of CVD and renal disease in CKD patients.

## Methods

### Study Population

The study protocol was approved by the ethics committee of the Third Affiliated Hospital of Sun Yat-sen University (Guangdong, China), and was approved by the Institutional Review Board. All methods were carried out in accordance with approved guidelines. Written informed consent was obtained from patients before enrollment. In this prospective cohort study, inpatients were recruited from July 2010 to December 2014. All information was extracted from our entire in-patient ABPM research database which was set up by our research group.

Inclusion criteria were patients: age ≥14 but <75 years; with CKD; who promised to return to the Third Affiliated Hospital of Sun Yat-sen University to complete the first follow-up (i.e., 6 months after the baseline survey).

Exclusion criteria were patients: with acute changes in the eGFR > 30% in the previous 3 months; undergoing dialysis; who had undergone kidney transplantation; with atrial fibrillation; with cardiovascular events in the previous 3 months; who were pregnant; had night-work or shift-work employment; could not tolerate ABPM; had invalid ABPM data.

697 CKD patients fulfilled inclusion criteria, 71 patients who fulfilled exclusion criteria were excluded and the detailed reasons could be found in Fig. 1. 38 patients were lost to follow-up after their first visit. Finally, 588 CKD patients were enrolled in this study.

### Measurements

#### Ambulatory Blood pressure monitoring

Patients underwent 24-h ABPM using a TM-2430 Monitor (A&D, Tokyo, Japan) as reported previously^23^,^24^. Briefly, cuff size was chosen based on arm circumference and applied to the non-dominant arm. BP was recorded every 15 min in daytime, and every 30 min at nighttime. Monitoring was done on a working day. Patients were asked to attend to their usual activities but to keep motionless at the time of measurement.

BP was measured for each patient during a visit to the physician. Sphygmomanometer measurements were recorded by the same physician, who was not aware of the results of ABPM recordings, as detailed elsewhere^23^,^24^. Briefly, measurements were taken in a quiet environment using a mercury sphygmomanometer with the patient in a sitting position after 5 min of rest. BP was not measured if the patient had consumed tobacco, ingested caffeine, or eaten within the previous 30 min. SBP and DBP values (Korotkoff’s phase I and phase V, respectively) at each visit enabled recording of a minimum of three BP measurements at intervals of ≥1 min.

#### Collection of other data

We collected urine samples from 7 AM to 7 AM the next day to identify the extent of proteinuria and sodium levels over 24 h. These patients were asked to void their bladders before and after urine collection. Proteinuria was measured by immunoturbidimetry. In addition, medical history, including demographic, laboratory data (hemoglobin, albumin, calcium, phosphorus, iPTH, serum fasting glucose, cholesterol, triglycerides, high-density lipoprotein cholesterol (HDL-C), low-density lipoprotein-cholesterol (LDL-C), homocysteine, uric acid, serum creatinine, BUN), and current therapy were obtained at the initial study visit. All experimental data were measured using a 7180 Biochemistry Auto-analyzer (Hitachi, Tokyo, Japan).

“ABPM daytime” and “ABPM nighttime” were defined according to patients’ schedules.

Participants with a reduction in SBP of ≥10% but <20% at nighttime compared with daytime were considered to have a dipping BP pattern. An extreme dipping BP pattern referred to a reduction at nighttime of >20%. A non-dipping BP pattern referred to a <10% reduction at nighttime. A reversed dipping BP pattern referred to higher SBP at nighttime compared with daytime^11^.

CKD was defined according to the KDIGO 2012 clinical practice guideline^25^. eGFR was calculated using 2009 CKD–EPI creatinine equation^26^.

DM was defined as the need for anti-DM drugs or meeting the diagnostic criteria based on the *Standards of Medical Care in Diabetes*^27^ set by the American Diabetes Association.

#### Outcomes

Primary endpoints were time to all-cause mortality and time to cardiovascular mortality. Secondary endpoints were time to renal events and time to cardiovascular events.

Cardiovascular mortality was defined as death caused by cardiovascular events. Renal events were a composite of doubling of serum creatinine or end-stage renal disease (ESRD), whichever occurred first. The endpoint of ESRD was reached on the day of the first dialysis session^28^. Cardiovascular events comprised fatal or non-fatal cardiovascular events: myocardial infarction, heart failure, revascularization, stroke, and other events (acute arterial occlusion of the lower extremities and thrombotic occlusion of the retinal artery), whichever occurred first. Cause of death was identified according to death certificates and autopsy reports based on the tenth revision of the *International Classification of Disease*. Hospital records were collected to establish the diagnosis based on criteria set by the American College of Cardiology and the European Society of Cardiology^29^,^30^. Patients were followed up until 31 March 2016, or death, and censored on the date of the last visit to the nephrology clinic.

### Statistical Analyses

Descriptive statistics are the mean ± SD for continuous variables and median values/interquartile range for nonparametric variables. Frequency and percentages are used for categorical variables.

Comparisons of continuous variables between groups were evaluated by the Student’s *t*-test, or nonparametric tests. Differences among categorical variables were analyzed using the χ^2^ test or Fisher’s exact test. Comparisons of continuous variables among groups were evaluated by ANOVA, or nonparametric tests. *P*-values for multiple comparisons were corrected according to the Bonferroni method.

We used STATA v14.0 to calculate the prevalence of endpoints. Crude rates and rates standardized by the direct method for sex and age are reported. Comparison of event rates among groups was done by the log-rank test. *P*-value for multiple comparisons was corrected according to the Bonferroni method.

We used Kaplan–Meier survival curves plotted according to current recommendations^31^, and the log-rank test to compare survival rate in different groups.

We used a competing risks model to compute hazard ratios associated with reverse dippers and non-dippers relative to dippers with regard to total mortality, cardiovascular mortality, renal events and cardiovascular events. In partly adjusted models, HRs were adjusted for age, sex, DM, cigarette smoking, alcohol consumption, body mass index, history of CVD, eGFR, hemoglobin level, phosphate level, cholesterol level, proteinuria and blockade of the renin–angiotensin system. In fully adjusted models, HRs were adjusted for these same parameters but also for 24-h BP.

All *P*-values were two-sided and the level of the test (α) was set as 0.05. Data were analyzed using SPSS *v*20.0 (IBM, Armonk, NY, USA) and STATA v14.0.

## Additional Information

**How to cite this article**: Wang, C. *et al*. Prognostic Value of Reverse Dipper Blood Pressure Pattern in Chronic Kidney Disease Patients not Undergoing Dialysis: Prospective Cohort Study. *Sci. Rep.* **6**, 34932; doi: 10.1038/srep34932 (2016).

## Figures and Tables

**Table 1:** Differences of baseline characteristics in Chinese non-dialysis CKD patients.

	Total (N = 588)	Dippers (N = 204)	Non-dippers (N = 256)	Reverse dippers (N = 106)	Extreme dippers (N = 22)	*P*-value
Age (years)	42.76 ± 16.71	40.19 ± 15.93	42.45 ± 17.10	48.55 ± 16.71^*^	42.36 ± 13.07	<0.001
Male: female ratio	336/252	122/82	151/105	51/55	12/10	0.209
Course (months)	6(1~24)	4(1~24)	6.0(1~24)	4.5(1~14.25)	10(1.75~36)	0.419
Diabetes mellitus (N/%)	92(15.6%)	22(10.8%)	37(14.4%)	27(25.5%)^*^	6(27.3%)	0.003
Current smoker (N/%)	109(18.5%)	37(18.2%)	46(18.0%)	21(19.8%)	5(22.7%)	0.941
Alcohol intake (N/%)	55(9.3%)	19(9.4%)	22(8.6%)	10(9.4%)	4(18.2%)	0.542
BMI (kg/m^2^)	23.16 ± 3.61	23.14 ± 3.59	23.10 ± 3.54	23.14 ± 3.73	24.22 ± 4.19	0.616
History of cardiovascular disease (N/%)	42(7.1%)	10(4.9%)	20(7.8%)	10(9.4%)	2(9.1%)	0.443
eGFR (mL/min per 1.73 m^2^)	67.84(29.83~106.69)	74.04(39.00~110.98)	66.65(23.99~104.93)	51.37(21.65~100.84)^*^	57.31(92.72~117.32)	0.004
Hemoglobin (g/L)	122.20 ± 24.15	127.11 ± 23.26	121.69 ± 24.23^*^	112.71 ± 23.89^*^	127.86 ± 19.36	<0.001
Albumin (g/L)	33.30 ± 9.02	34.41 ± 9.14	32.65 ± 9.14	31.92 ± 8.30	37.23 ± 8.16	0.011
Total calcium level (mmol/L)	2.19 ± 0.20	2.21 ± 0.20	2.19 ± 0.21	2.15 ± 0.17^*^	2.29 ± 0.17	0.006
Phosphate level (mmol/L)	1.26 ± 0.24	1.25 ± 0.21	1.28 ± 0.26	1.25 ± 0.24	1.15 ± 0.21	0.101
iPTH (pg/mL)	51.87 (31.56~87.89)	44.18(28.64~72.82)	57.80(36.42~108.68)^*^	54.22(34.65~86.92)	32(24.52~54.69)	0.011
Serum fasting glucose (mmol/L)	5.09 ± 1.44	5.05 ± 1.23	5.05 ± 1.44	5.30 ± 1.83	5.01 ± 1.13	0.464
Cholesterol (mmol/L)	6.15 ± 2.96	6.09 ± 2.91	6.27 ± 3.07	6.13 ± 3.01	5.43 ± 1.65	0.619
Triglyceride (mmol/L)	2.08 ± 1.78	1.95 ± 1.19	2.24 ± 2.25	2.06 ± 1.51	1.67 ± 1.16	0.254
HDL-C (mmol/L)	1.23 ± 0.42	1.23 ± 0.42	1.25 ± 0.43	1.19 ± 0.41	1.30 ± 0.52	0.485
LDL-C (mmol/L)	4.03 ± 2.32	4.09 ± 2.24	4.00 ± 2.40	4.05 ± 2.42	3.70 ± 1.51	0.896
Uric acid (mmol/L)	444.60 ± 130.67	440.35 ± 128.85	451.81 ± 139.30	447.75 ± 114.49	385.16 ± 103.30	0.132
Blood urea nitrogen (mmol/L)	6.80(4.93~11.13)	6.08(4.70~8.80)	7.72(5.10~13.01)^*^	7.62(5.40~12.73)^*^	5.71(4.26~7.41)	<0.001
Serum creatinine (μmol/L)	107.2(71.90~192.00)	98.20(69.05~153.62)	107.75(73.32~228.37)	133.0(72.7~244.0)^*^	78.65(58.75~128.10)	0.018
Homocysteine (μmol/L)	15.60 ± 8.31	14.22 ± 6.80	17.67 ± 10.00^*^	14.12 ± 6.14	14.13 ± 6.58	<0.001
Urinary sodium excretion (mmol/24 h)	130.69 ± 80.60	127.70 ± 73.30	140.81 ± 90.10	110.17 ± 56.82	130.00 ± 98.81	0.098
Proteinuria (g/24 h)	1.73(0.46~4.55)	1.39(0.39~3.87)	1.94(0.46~5.04)	2.09(0.96 ~5.36)^*^	0.55(0.24~3.59)	0.005
Clinic-SBP (mmHg)	140.25 ± 22.92	138.20 ± 22.22	140.56 ± 23.13	144.13 ± 24.48	136.82 ± 17.24	0.297
Clinic-DBP (mmHg)	85.38 ± 13.23	84.09 ± 13.07	86.03 ± 13.58	86.67 ± 13.25	83.59 ± 9.49	0.265
24 h-SBP (mmHg)	129.05 ± 17.24	125.38 ± 15.41	129.80 ± 17.53^*^	135.04 ± 18.54^*^	125.45 ± 15.13	<0.001
24 h-DBP (mmHg)	77.80 ± 10.03	76.17 ± 9.72	78.26 ± 9.86	80.11 ± 10.85^*^	76.36 ± 8.42	0.007
SBP-daytime (mmHg)	130.61 ± 17.19	128.23 ± 15.80	131.06 ± 17.71	134.15 ± 18.31^*^	130.45 ± 15.53	0.035
DBP-daytime (mmHg)	78.96 ± 10.10	78.02 ± 10.04	79.25 ± 9.95	79.96 ± 10.86	79.50 ± 8.40	0.380
SBP-nighttime (mmHg)	121.00 ± 19.57	110.76 ± 13.70	123.43 ± 16.79^*^	139.36 ± 19.95^*^	99.27 ± 13.95^*^	<0.001
DBP-nighttime (mmHg)	71.77 ± 11.56	66.42 ± 9.32	73.14 ± 10.12^*^	81.19 ± 11.62^*^	60.09 ± 9.37^*^	<0.001
Receiving no antihypertensive drugs	147(25.0%)	47(23.0%)	76(29.7%)	24(22.6%)	0(0%)	0.012
Bedtime dosing of hypertensive drugs	118(20.1%)	40(19.6%)	49(19.1%)	21(19.8%)	8(36.4%)	0.394
On RAS blockade	341(58.0%)	123(60.6%)	142(55.5%)	55(51.9%)	21(95.4%)*	0.002
Calcium-channel blocker	196(33.3%)	67(33.0%)	79(30.9%)	44(41.5%)	6(27.3%)	0.152
α-blocker	46(7.8%)	15(7.4%)	20(7.8%)	10(9.4%)	1(4.5%)	0.821
β-blocker	94(16.0%)	33(16.3%)	35(13.7%)	25(23.6%)	1(4.5%)	0.037
Statins	103(17.5%)	39(19.2%)	45(17.6%)	17(16.0%)	2(9.1%)	0.696

BMI: body mass index; CKD: chronic kidney disease; DBP: diastolic blood pressure; eGFR: estimated glomerular filtration rate; HDL-C: high-density lipoprotein cholesterol; iPTH: intact parathyroid hormone; LDL-C: low-density lipoprotein cholesterol; RAS: renin-angiotensin system; SBP: systolic blood pressure.

*P*-value for analysis of variance or χ^2^ test between 4 groups.

Choosing dippers as reference group, and comparing the 3 other groups (non-dipper, reverse-dipper, extreme dipper) to this group throughout. * indicated comparison with the dippers *P* < 0.017.

**Table 2:** Incidence of events by blood pressure pattern.

	Dippers	Non-dippers	Reverse dippers
Total mortality			
Crude rate	10.90(2.87~18.93)	19.65(13.17~26.13)	69.13(39.90~98.36)^*^
Standardized rate	13.06(4.28~21.84)	17.6(8.27~26.93)	56.77(30.11~83.43)^*^
Cardiovascular mortality			
Crude rate	9.28(1.89~16.67)	18.18(11.96~24.40)	58.76(31.66~85.86)^*^
Standardized rate	10.72(2.78~18.66)	16.36(7.40~25.32)	49.08(24.19~73.97)^*^
Renal events			
Crude rate	41.06(25.30~56.82)	108.15(92.90~123.40)^*^	158.38(112.78~203.98)^*^
Standardized rate	42.03(26.09~57.97)	101.01(78.28~123.74)^*^	132.38(90.05~174.71)^*^
Cardiovascular events			
Crude rate	15.59(6.00~25.18)	45.65(35.78~55.52)^*^	101.38(65.13~137.63)^*^
Standardized rate	16.31(6.51~26.11)	41.94(27.54~56.34)^*^	82.71(49.63~115.79)^*^

Values are rates (95% confidence interval), expressed as number of events per 1000 patient-years. Rates are crude or standardized for sex, age by the direct method. Comparison of event rates among groups was done by log-rank test.

Choosing dippers as reference group, and comparing the 2 other groups (non-dipper, reverse-dipper) to this group throughout. *Indicated comparison with the dippers *P* < 0.025.

**Table 3:** Risk of total mortality, cardiovascular mortality, renal events and cardiovascular events in reverse dippers.

Characteristic	Total mortality	Cardiovascular mortality	Renal events	Cardiovascular events
Non-dippers (*versus* dippers)				
Not adjusted	1.83(0.75~4.49),*P* = 0.187	2.00(0.76~5.24),*P* = 0.158	2.62(1.66~4.13),*P* < 0.001	2.88(1.42~5.83),*P* = 0.003
Partly adjusted	1.01(0.33~3.11),*P* = 0.990	1.04(0.28~3.90),*P* = 0.95	1.92(1.09~3.37),*P* = 0.024	1.96(0.86~4.47),*P* = 0.108
Fully adjusted	0.71(0.22~2.29),*P* = 0.568	0.64(0.18~2.33),*P* = 0.499	1.77(0.99~3.14),*P* = 0.053	1.60(0.70~3.64),*P* = 0.261
Reverse dippers (*versus* dippers)				
Not adjusted	6.41(2.71~15.17),*P* < 0.001	6.24(2.46~15.84),*P* < 0.001	3.68(2.22~6.11),*P* < 0.001	6.21(3.01~12.83),*P* < 0.001
Partly adjusted	6.36(2.30~17.62),*P* < 0.001	5.39(1.67~17.42),*P* = 0.005	3.34(1.81~6.14),*P* < 0.001	4.75(2.05~11.00),*P* < 0.001
Fully adjusted	5.08(1.79~14.47),*P* = 0.002	4.17(1.25~13.88),*P* = 0.02	3.00(1.59~5.65),*P* = 0.001	4.12(1.78~9.51),*P* = 0.001

Data are hazard ratios (95% CI), followed by the *P* value, which express the risk of non-dippers (*versus* dippers) and reverse dippers (*versus* dippers).

Partly adjusted hazard ratios were adjusted for age, gender, diabetes mellitus, smoking and drinking, body-mass index, history of cardiovascular disease, eGFR, hemoglobin, phosphate, cholesterol, proteinuria and on RAS blockade.

In fully adjusted models, they were additionally adjusted for 24 h blood pressure.
